# The association of polymorphisms in hormone metabolism pathway genes, menopausal hormone therapy, and breast cancer risk: a nested case-control study in the California Teachers Study cohort

**DOI:** 10.1186/bcr2859

**Published:** 2011-04-01

**Authors:** Eunjung Lee, Fredrick Schumacher, Juan Pablo Lewinger, Susan L Neuhausen, Hoda Anton-Culver, Pamela L Horn-Ross, Katherine D Henderson, Argyrios Ziogas, David Van Den Berg, Leslie Bernstein, Giske Ursin

**Affiliations:** 1 Department of Preventive Medicine, Keck School of Medicine, University of Southern California, 1441 Eastlake Avenue, Los Angeles, CA 90089, USA; 2 Department of Population Sciences, Beckman Research Institute, City of Hope, 1500 Duarte Road, Duarte, CA 91010, USA; 3 Department of Epidemiology, School of Medicine, University of California, 224 Irvine Hall, Irvine, CA 92697-7550, USA; 4 Cancer Prevention Institute of California, 2201 Walnut Avenue, Fremont, CA 94538, USA; 5 Department of Nutrition, University of Oslo, Sognsvannsveien 9, 0372 Oslo, Norway; 6 Cancer Registry of Norway, PB 5313, Majorstuen, 0304 Oslo, Norway

## Abstract

**Introduction:**

The female sex steroids estrogen and progesterone are important in breast cancer etiology. It therefore seems plausible that variation in genes involved in metabolism of these hormones may affect breast cancer risk, and that these associations may vary depending on menopausal status and use of hormone therapy.

**Methods:**

We conducted a nested case-control study of breast cancer in the California Teachers Study cohort. We analyzed 317 tagging single nucleotide polymorphisms (SNPs) in 24 hormone pathway genes in 2746 non-Hispanic white women: 1351 cases and 1395 controls. Odds ratios (ORs) and 95% confidence intervals (CIs) were estimated by fitting conditional logistic regression models using all women or subgroups of women defined by menopausal status and hormone therapy use. *P *values were adjusted for multiple correlated tests (*P*_ACT_).

**Results:**

The strongest associations were observed for SNPs in *SLCO1B1*, a solute carrier organic anion transporter gene, which transports estradiol-17β-glucuronide and estrone-3-sulfate from the blood into hepatocytes. Ten of 38 tagging SNPs of *SLCO1B1 *showed significant associations with postmenopausal breast cancer risk; 5 SNPs (rs11045777, rs11045773, rs16923519, rs4149057, rs11045884) remained statistically significant after adjusting for multiple testing within this gene (*P*_ACT _= 0.019-0.046). In postmenopausal women who were using combined estrogen-progestin therapy (EPT) at cohort enrollment, the OR of breast cancer was 2.31 (95% CI = 1.47-3.62) per minor allele of rs4149013 in *SLCO1B1 *(*P *= 0.0003; within-gene *P*_ACT _= 0.002; overall *P*_ACT _= 0.023). SNPs in other hormone pathway genes evaluated in this study were not associated with breast cancer risk in premenopausal or postmenopausal women.

**Conclusions:**

We found evidence that genetic variation in *SLCO1B1 *is associated with breast cancer risk in postmenopausal women, particularly among those using EPT.

## Introduction

Reproductive and hormonal factors, including age at menarche, parity, number of full-term pregnancies, age at first full-term pregnancy, breastfeeding, age at menopause, body mass index (BMI), and physical activity, are associated with breast cancer risk [1,2]. Consistent with these observations, breast cancer risk is higher among women with higher circulating levels of endogenous estrogen [3-5] and among women using combined postmenopausal estrogen and progestin therapy (EPT) [6-11].

Sex steroid hormones, whether endogenous or exogenous, are synthesized and metabolized by many different enzymes (reviewed in [12]). Therefore, genetic variation among genes regulating sex steroid hormone levels may increase or decrease breast cancer risk by influencing hormone metabolism. Polymorphisms in several hormone pathway genes, including *CYP19A1 *and *COMT*, have been associated with endogenous hormone levels [13-16]; however, studies investigating the association of genetic variation in hormone metabolism genes and breast cancer risk have generated mixed results [14,17-29]. Recently, a large study from the Breast and Prostate Cancer Cohort Consortium (BPC3) comprehensively analyzed 37 steroid hormone metabolism pathway genes in relation to breast cancer risk and reported null associations [16,30], suggesting that inconsistencies in the literature may be due to findings observed by chance in small studies. However, it is possible that the inconsistencies may be explained at least partly by differences in the distribution of environmental factors that modify the effects of genetic polymorphisms.

EPT use increases breast cancer risk to a much greater extent than estrogen-only therapy (ET) [6-11]. Therefore, it is important to examine EPT and ET use separately when investigating gene-hormone therapy (HT) interactions. To date, few studies have investigated the gene-HT interactions by hormone formulation by using a comprehensive single-nucleotide polymorphism (SNP)-tagging approach. The California Teachers Study is an effective resource to study these questions because detailed data on hormone use were collected at baseline, and approximately 41% and 28% of the postmenopausal participants reported current use of EPT and ET, respectively [31]. Thus, using data from a case-control study nested within the California Teachers Study, we systematically investigated whether any of 24 hormone metabolism pathway genes or their interactions with HT were associated with breast cancer risk.

## Materials and methods

### Participants

The California Teachers Study has been previously described in detail [32]. Briefly, the California Teachers Study is a prospective cohort of women who were current, recent, or retired California public school professionals in 1995. By returning a baseline questionnaire in 1995-1996, 133,479 women joined the cohort and provided detailed information on menopausal status, HT use, and other lifestyle and medical factors. The baseline questionnaire is available on the California Teachers Study website [33]. Cancer diagnoses in the cohort are identified through annual linkage with the California Cancer Registry, which identifies at least 99% of cancers diagnosed in California [34]. The California Teachers Study has been approved by the institutional review boards at all participating institutions: the Cancer Prevention Institute of California (CPIC), the University of California at Irvine (UCI), the University of Southern California (USC), and the City of Hope in accordance with assurances filed with and approved by the US Department of Health and Human Services.

The nested breast cancer case-control study was designed to obtain biospecimens from breast cancer cases and unaffected controls within the 113,590 members of the cohort who were less than 80 years old at baseline, had continued residence in California during the study period (1995 to time of blood draw), and, before 1998, had no prior history of invasive or *in situ *breast cancer. Cases were women who had a histologically confirmed invasive primary carcinoma of the breast (International Classification of Disease for Oncology code C50 restricted to morphology codes under 8,590) and who were 80 years old or younger between 1 January 1998 and 31 May 2007. One control participant per case was randomly selected from the cohort and frequency-matched to the case on age at baseline (within 5-year age groups), self-reported race/ethnicity (white, African-American, Latina, Asian, and other), and three broad geographic regions (that is, California Teachers Study specimen collection centers). Cancer cases were identified through quarterly linkages of the cohort to the California Cancer Registry database. Control selection was conducted on a quarterly basis without replacement. For each wave of control selection, a reference date was determined (that is, January, April, July, and October of each year). Nearly equal numbers of controls were selected in each wave. One control participant had her breast cancer diagnosed after her control selection and was included in the analysis as both a control and a case.

### Collection of biological specimens and DNA extraction

Collection of biological specimens was conducted at three study centers (CPIC in the northern half of California and USC and UCI in the southern half). Women who declined blood draw were asked whether they were willing to provide a saliva sample, and, if so, an Oragene DNA self-collection kit (DNA Genotek, Kanata, ON, Canada) was mailed to the participant with informed consent and return postage paid mailing materials. From the 8,118 eligible participants (2,618 cases and 5,500 selected controls), we collected biological specimens for 74% of the cases (1,923 cases: 1,684 blood specimens and 239 saliva specimens) and 61% of the controls (3,350 controls: 3,012 blood specimens and 338 saliva specimens). All biologic samples were sent via overnight courier to the UCI laboratory for DNA extraction. DNA was extracted from blood clots by using Qiagen Clotspin Baskets and DNA QIAmp DNA Blood maxi kits (Qiagen Inc., Valencia, CA, USA) in accordance with Qiagen protocols. DNA was extracted from saliva samples by using the Oragene protocol (DNA Genotek). The nested breast cancer case-control study of the California Teachers Study has been approved by the institutional review boards at all participating institutions, and all participants provided written informed consent.

### Tagging SNP selection and genotyping

We investigated 24 genes that are involved in female sex steroid hormone biosynthesis, metabolism, or excretion. Reviews on the function of these genes are available elsewhere [12,35,36]. For 21 of these genes, a tagging SNP approach was used (Supplementary Table S1 in Additional file 1). For 16 of the 21 genes, we selected linkage disequilibrium tagging SNPs across each gene, 20 kb upstream of 5' untranslated region (UTR) and 10 kb downstream of 3' UTR. The tagging SNPs were selected to capture all common SNPs (minor allele frequency (MAF) of at least 5%) in individuals of European ancestry with minimum pairwise R^2 ^of at least 0.80 by using the Snagger software [37] and the data from the International HapMap Project for the white CEPH (Utah residents with ancestry from northern and western Europe) population (HapMap release 21, July 2006, genotype build 36 [38]). Tagging SNPs for five genes included in the present study had been selected by BPC3 by using the TagSNPs program [30,39]. To facilitate the comparison across studies, we used the BPC3-selected tagging SNPs for these five genes (Supplementary Table S1 in Additional file 1). The BPC3-selected tagging SNPs captured all common SNPs (MAF of at least 5%) in whites with minimum pairwise R^2 ^of at least 0.8. For the remaining 3 of the 24 genes, we genotyped a few selected SNPs due to space limitations of our genotyping platform. For CYP19A1, the selected SNPs were shown to be associated with circulating estrogen concentrations in a comprehensive analysis [13] (Supplementary Table S1 in Additional file 1).

A total of 1,751 breast cancer cases and 1,697 controls were available for genotyping. We included a random sample of 193 replicates (105 cases and 88 controls) to monitor reproducibility and track plate flips or switches. The DNA samples were genotyped for the selected tagging SNPs by using the Illumina Golden Gate Assay (Illumina, Inc., San Diego, CA, USA) in the USC Core Facility. About 10% of the genotyped samples, including 189 cases and 150 controls, had a genotyping success rate (call rate) of less than 90% and were excluded from the analyses. The genotyping concordance rate based on the 160 duplicate samples with a call rate of at least 90% was 99.9%. Call rates were lower when the DNA was obtained from saliva samples: 23% of saliva samples and 8% of blood samples had a call rate of less than 90% and these samples therefore were excluded from the analyses. However, the genotyping concordance after excluding the low call rate samples was excellent for saliva samples (greater than 99.9%). In addition, results from sensitivity analyses excluding saliva samples were similar to those using all samples. For the present study, we also excluded 88 women (52 cases and 36 controls) who self-reported to have had a previous history of cancer, leaving 1,510 cases and 1,511 controls. Because the majority (approximately 91%) of participants were non-Hispanic whites, we restricted the analyses to 2,746 non-Hispanic white women (1,351 cases and 1,395 controls).

Of the 355 SNPs genotyped, 332 had an SNP call rate of at least 90%. We excluded an additional three SNPs that had discordant readings in more than two duplicate pairs, eight SNPs with an MAF of less than 1% among non-Hispanic white controls, and four SNPs in *COMT, CYP11A, SULT1A1; SULT1A2, UGT1A8 *not in Hardy-Weinberg equilibrium (*P *< 0.001); thus, we analyzed 317 SNPs in the present study.

For 19 of the 20 genes for which we used the tagging SNP approach, the genotyped tagging SNPs efficiently captured 70% to 100% of all common SNPs (MAF of greater than 5%) in the HapMap dataset of European ancestry (HapMap release 24, genotype build 36) with pairwise R^2 ^of at least 0.80 (Supplementary Table S1 in Additional file 1). We did not have sufficient tagging coverage for CYP21A2.

### Imputation

We imputed SNPs in gene regions where we found a statistically significant association (*P *< 0.01 before multiple testing correction) with breast cancer risk among all women or among subgroups defined by menopausal status. To do this, we used publicly available HapMap genotype data in the CEPH population as the reference sample (HapMap release 24, genotype build 36 [38]) and MACH 1.0 [40]. We excluded imputed SNPs when the MAF was less than 1% or when the R^2 ^was less than 0.30 [40].

### Statistical analyses

We used conditional logistic regression models with strata defined by 5-year age group and the three specimen collection centers to estimate the odds ratios (ORs), 95% confidence intervals (CIs), and *P *values associated with each SNP by using log-additive models. The results did not change after further adjustment for potential confounders including menopausal status (premenopausal, postmenopausal, and unknown), HT use status at baseline (never used HT, currently using ET, currently using EPT, used HT in the past, and unknown), BMI (less than 25, 25 to less than 30, at least 30 kg/m^2^, and unknown), parity (0, 1 to 2, at least 3, and unknown), and oral contraceptive use (never, ever, and unknown). Therefore, we presented the results from the conditional logistic regression models not adjusting for these potential confounders.

We performed subgroup-specific analyses by menopausal status and, among postmenopausal women, by HT use at baseline, defining the groups of interest as never used HT, currently using ET, and currently using EPT. We calculated *P *for interaction by likelihood ratio test comparing the model with and without the product term of genotype (0, 1, and 2 copies of minor allele, as ordinal variable) and menopausal status or HT use. The interaction tests for HT use were done separately for current EPT use (as compared with never HT use) and for current ET use (as compared with never HT use).

## Results

A greater proportion of breast cancer cases than controls were currently using EPT at baseline; a lower proportion of cases than controls had high parity. Cases also had slightly earlier age at menarche than control women (Table 1).

**Table 1:** Characteristics of the study participants at time of joining the cohort

	Control		Case	
	
Characteristics	Number	Percentage	Number	Percentage
Number	1,395		1,351	
Mean age ± SD, years	56.1 ± 9.5		55.0 ± 9.4	
Menopausal status				
Premenopausal	347	25.3	364	27.5
Postmenopausal^a^	1,024	74.7	962	72.5
Unknown	24	-	25	-
HT use among postmenopausal women				
Never used HT	153	15.8	112	12.4
Current ET use	277	28.7	230	25.5
Current EPT use	401	41.5	468	51.9
Former ET or EPT use	122	12.6	80	8.9
Ever used progestin alone	13	1.3	12	1.3
Unknown	58	-	60	-
Parity/total number of FTPs				
Nulligravid	221	16.1	219	16.4
Gravid, nulliparous	61	4.4	66	4.9
1 FTP	159	11.6	174	13.0
2 FTPs	487	35.4	472	35.3
3 FTPs	274	20.0	277	20.7
4+ FTPs	172	12.5	128	9.6
Unknown	21	-	15	-
Body mass index				
< 25 kg/m^2^	765	54.8	778	58.9
25 to < 30 kg/m^2^	387	27.7	384	29.1
30+ kg/m^2^	195	14.0	159	12.0
Unknown	48	-	30	-
Age at menarche				
≤10 years	100	7.3	100	7.5
11-12 years	600	43.6	599	44.8
13-14 years	565	41.0	542	40.6
15-16 years	102	7.4	82	6.1
17+ years	10	0.7	13	1.0
Unknown/Never had menarche	18	-	15	-
Family history of breast cancer (first-degree relative)				
No	1,167	86.1	1,085	82.1
Yes	189	13.9	236	17.9
Unknown	39	-	30	-
History of breast biopsy				
No	1,086	77.8	1,034	76.5
Yes	309	22.2	317	23.5
Screening mammograms within last 2 years				
No	138	10.0	146	10.9
Yes	1,241	90.0	1,193	89.1
Unknown	16	-	12	-

^a^Includes perimenopausal women. EPT, estrogen progestin combined therapy; ET, estrogen therapy; FTP, full-term pregnancy; HT, hormone therapy; SD, standard deviation.

Evaluation of the q-q plot of the *P *values for the association between the 317 SNPs in the 24 hormone metabolism genes and breast cancer risk showed no evidence of systematic bias (Supplementary Figure S1 in Additional file 2).

We observed statistically significant associations at a *P *value of less than 0.01 with two SNPs in *SLCO1B1 *and one SNP in *HSD17B4 *in the overall analysis (Table 2). However, after multiple comparisons were corrected for, none of these associations was statistically significant. For postmenopausal women, 10 SNPs in *SLCO1B1 *were associated with breast cancer risk with an uncorrected *P *value of less than 0.01. Of these, the associations for SNPs rs11045773, rs11045777, rs16923519, rs4149057, and rs11045884 remained statistically significant after correction for multiple testing within the gene (within-gene P_ACT _< 0.05). However, after multiple testing across all genes was corrected for, none of these associations was statistically significant. The ORs and 95% CIs associated for all tested SNPs are provided in Supplementary Table S2 in Additional file 3. There was some evidence that, of these, rs4149013 in *SLCO1B1 *was associated with breast cancer risk in postmenopausal women (OR 1.39, 95% CI 1.07 to 1.81; uncorrected *P *= 0.015).

**Table 2:** Single-nucleotide polymorphisms associated with breast cancer risk with *P *values of less than 0.01^a^

	All subjects						Premenopausal						Postmenopausal						
		
Locus	WW ca/co	WV ca/co	VV ca/co	OR (95% CI)	P	P_ACT_^b^	WW ca/co	WV ca/co	VV ca/co	OR (95% CI)	P	P_ACT_^b^	WW ca/co	WV ca/co	VV ca/co	OR (95% CI)	P	P_ACT_^b^	P int^†^
SLCO1B1																			
rs11045773	898/857	401/468	50/67	0.83 (0.72-0.94)	0.0047	0.088	227/206	118/127	18/13	0.94 (0.72-1.21)	0.61	1.00	659/636	273/332	29/54	0.77 (0.65-0.90)	0.0009	0.020	0.17
rs11045777	899/855	401/471	50/64	0.83 (0.73-0.95)	0.0053	0.099	227/204	118/129	18/11	0.94 (0.73-1.22)	0.66	1.00	660/636	273/333	29/53	0.77 (0.65-0.90)	0.0009	0.019	0.17
rs976754	1,126/1,207	217/183	8/5	1.27 (1.03-1.55)	0.018	0.26	309/294	50/51	5/2	1.04 (0.71-1.53)	0.76	1.00	795/894	164/127	3/3	1.40 (1.10-1.79)	0.0046	0.077	0.15
rs16923519	836/921	452/421	62/53	1.17 (1.02-1.33)	0.018	0.26	244/222	107/114	12/11	0.92 (0.70-1.20)	0.53	1.00	577/685	335/297	50/42	1.26 (1.08-1.47)	0.0019	0.038	0.029
rs4149057	496/569	641/638	209/186	1.14 (1.02-1.27)	0.021	0.28	152/133	164/172	48/42	0.96 (0.77-1.19)	0.70	1.00	334/429	466/453	157/140	1.22 (1.07-1.39)	0.0024	0.046	0.047
rs4149058	778/854	468/467	95/72	1.15 (1.02-1.30)	0.029	0.34	220/204	122/120	21/22	0.94 (0.73-1.20)	0.66	1.00	541/636	342/337	71/50	1.23 (1.07-1.43)	0.0043	0.074	0.052
rs11045884	880/871	412/452	41/63	0.86 (0.75-0.99)	0.031	0.36	216/220	126/107	15/16	1.09 (0.84-1.42)	0.42	1.00	649/638	277/335	25/46	0.78 (0.66-0.92)	0.0023	0.044	0.033
rs11045825	953/943	357/394	38/55	0.87 (0.75-1.00)	0.045	0.45	237/236	112/97	14/13	1.09 (0.83-1.42)	0.54	1.00	700/692	237/288	23/42	0.79 (0.66-0.93)	0.0040	0.068	0.037
rs10841767	889/874	411/451	49/67	0.88 (0.77-1.00)	0.049	0.47	221/222	125/107	17/18	1.07 (0.83-1.38)	0.51	1.00	653/639	277/334	31/48	0.80 (0.69-0.94)	0.0059	0.091	0.058
rs11045813	917/918	372/401	37/56	0.88 (0.76-1.01)	0.068	0.58	225/229	118/99	13/12	1.12 (0.85-1.47)	0.40	1.00	677/676	245/293	23/44	0.79 (0.67-0.94)	0.0051	0.082	0.024
HSD17B4																			
rs382719	393/457	672/685	275/241	1.16 (1.04-1.29)	0.0084	0.097	105/124	179/172	76/51	1.32 (1.06-1.64)	0.012	0.12	282/329	479/499	194/184	1.11 (0.98-1.26)	0.11	0.657	0.16
rs17388769	919/1,002	393/364	33/21	1.21 (1.04-1.41)	0.012	0.13	243/258	108/89	11/0	1.56 (1.15-2.13)	0.004	0.051	656/729	280/266	22/21	1.12 (0.94-1.34)	0.15	0.77	0.074

^a^Single-nucleotide polymorphisms associated with breast cancer risk with *P *values of less than 0.01 (uncorrected for multiple testing) in either overall analyses or subgroup analyses of premenopausal or postmenopausal women. The odds ratios (ORs) and 95% confidence intervals (CIs) were based on conditional logistic regression models stratified by age group (within 5-year age groups) and specimen collection centers - the Cancer Prevention Institute of California, the University of Southern California, and the University of California at Irvine - using log-additive genetic models. ^b^P_ACT _(*P *value adjusted for correlated tests) within each gene was calculated by using the methods of Conneely and Boehnke [68]. When P_ACT _values were applied across all genes evaluated, none of the values was statistically significant. ^c ^*P *values for interaction.

CI, confidence interval; OR, odds ratio; SNP, single-nucleotide polymorphism.

When examining by HT use, we observed a strong association between several SNPs in *SLCO1B1 *and postmenopausal breast cancer risk in current EPT users (Table 3). Breast cancer risk among postmenopausal women who were using EPT at baseline increased more than twofold per minor allele of rs4149013 (OR 2.31, 95% CI 1.47 to 3.62; *P *= 0.0003, within-gene P_ACT _= 0.002). This association was statistically significant even after P_ACT _adjustment across all SNPs studied (P_ACT _= 0.023). The *P *value for interaction (EPT versus never HT use) was 0.019 (not corrected for multiple testing). When we combined the homozygous and heterozygous minor allele carriers (that is, a dominant genetic model), we observed similar OR estimates and *P *values (OR 2.43, 95% CI 1.53 to 3.85; *P *= 0.0002) (Supplementary Table S3 in Additional file 4). We did not observe any significant associations in never HT users and ET users. There was no statistically significant difference in effects when stratifying by estrogen receptor status.

**Table 3:** Single-nucleotide polymorphisms associated with breast cancer risk in postmenopausal women using estrogen-progestin therapy at baseline^a^

		Never used HT			Using ET				Using EPT			
		
Gene	SNP	N^b^ (ww/wv/vv)	OR (95% CI)	P^c^	N^b^ (ww/wv/vv)	OR (95% CI)	P^c^	P_int_^d^	N^b^ (ww/wv/vv)	OR (95% CI)	P^c^ **(P_ACT_^e^; P_ACT_^f^**)	P_int_^d^
SLCO1B1	rs4149013	114/12/1 148/20/1	0.79 (0.39-1.60)	0.51	199/31/0 245/31/0	1.20 (0.69-2.03)	0.54	0.51	393/71/1 373/27/1	2.31 (1.47-3.62)	0.0003 (0.002; 0.023)	0.019
SLCO1B1	rs976754	110/16/1 143/25/1	0.87 (0.45-1.67)	0.67	198/32/0 246/31/0	1.21 (0.71-2.05)	0.48	0.51	375/91/2 351/49/1	1.71 (1.19-2.47)	0.004 (0.045; 0.37)	0.11
SLCO1B1	rs11045777	86/35/6 104/56/8	0.68 (0.42-1.09)	0.11	155/66/9 180/82/14	0.88 (0.64-1.19)	0.40	0.42	327/131/10 248/134/19	0.72 (0.56-0.92)	0.009 (0.10; 0.68)	0.95
SLCO1B1	rs11045773	86/35/6 103/56/9	0.66 (0.41-1.06)	0.09	155/66/9 181/81/14	0.88 (0.65-1.20)	0.42	0.36	326/131/10 248/134/19	0.72 (0.56-0.92)	0.009 (0.11; 0.70)	0.86

^a^single-nucleotide polymorphisms that were associated with breast cancer risk with *P *values of less than 0.01 (uncorrected for multiple testing) in subgroup analyses of postmenopausal women who were using estrogen-progestin therapy at baseline. The odds ratios (ORs) and 95% confidence intervals (CIs) were based on conditional logistic regression models stratified by age group (within 5-year age groups) and specimen collection centers - the Cancer Prevention Institute of California, the University of Southern California, and the University of California at Irvine - using log-additive genetic models. ^b^Number of cases carrying 0, 1, and 2 copies of minor allele and number of controls carrying 0, 1, and 2 copies of minor allele, respectively. ^c^*P *values not corrected for multiple testing. ^d^*P *values for interaction with never hormone therapy (HT) users. ^e^P_ACT _(*P *value adjusted for correlated tests) was calculated within each gene by using the methods of Conneely and Boehnke [68]. ^f^P_ACT _was calculated across all genes by using the methods of Conneely and Boehnke [68]. CI, confidence interval; EPT, estrogen-progestin therapy; ET, estrogen-only therapy; OR, odds ratio; SNP, single-nucleotide polymorphism.

## Discussion

In this case-control study nested within the California Teachers Study cohort, genetic variation in only 1 (*SLCO1B1*) of 24 genes in the hormone metabolism pathway genes was associated with breast cancer risk. *SLCO1B1*, a gene involved in the hepatic uptake of female sex steroids, seemed to be associated with breast cancer risk among postmenopausal women. This association was statistically significant after correcting for multiple testing within the gene but was not statistically significant after we corrected for multiple testing across genes. However, there was also an indication that EPT may interact with SNPs in *SLCO1B1*; one variant in *SLCO1B1 *(rs4149013) was statistically significantly associated with breast cancer risk in EPT users.

Our findings of no association between SNPs in hormone metabolism pathway genes and breast cancer risk are consistent with the results from other large studies such as BPC3 [13,29,30,41] and meta-analyses of selected functional SNPs in *CYP1A1 *[23], *SULT1A1 *[42,43], *CYP1B1 *[44,45], and *COMT *[46], although two smaller meta-analyses of selected functional SNP in *COMT *supported an association in Caucasian populations [47,48]. Although a few studies have suggested associations between genetic polymorphisms in other hormone pathway genes, including *CYP11A *[25,26], *CYP1A1*/*CYP1A2 *[49,50], *CYP1B1 *[51,52], *SULT1E1 *[53], or *COMT *[54], many of these associations were not observed consistently [30,49,50,55-57]. In addition, the few studies other than BPC3 that have investigated polymorphisms in *CYP2C9 *[51], *CYP3A4 *[49,51,56], *HSD17B2 *[58], *SRD5A1 *[56], and *UGT2B7 *[51], in relation to breast cancer risk among Caucasian populations, have reported no associations. However, a recent study using admixture maximum likelihood (AML)-based global tests reported that genetic variation in androgen-estrogen conversion pathway was associated with breast cancer risk, although no single SNP was significant after correcting multiple testing [59].

To our knowledge, no studies have investigated genetic variation in *SLCO1B1 *and breast cancer risk by hormone therapy use. *SLCO1B1*, also known as *OATP-C *or *SLC21A6*, is expressed in the liver and plays an important role in transporting drugs and endogenous substrates from the blood into the hepatocytes (reviewed in [60]). Endogenous substrates of SLCO1B1 include steroid hormone conjugates such as estradiol-17β-glucuronide and estrone-3-sulfate [61,62]. Serum estrone sulfate (E1S) is a major form of circulating estrogen in postmenopausal women and can be converted to estradiol in breast tissue [63]. E1S is also a major component of conjugated equine estrogens, the estrogen component of the predominant (prior to 2002) HT regimens in the US [64]. Genetic variation in *SLCO1B1 *has been shown to decrease the uptake of E1S and estradiol glucuronide in several [61,65] but not all [66] studies. Furthermore, one study has shown that genetic variation in *SLCO1B1 *is associated with blood E1S levels in Caucasians [61], suggesting that genetic variation in *SLCO1B1 *may interact with HT use. Rs4149013 is located near the 5' end of *SLCO1B1*. The functional significance of this variant is not known, but even if there is none, this variant could be linked to a causal allele.

In the publicly available Cancer Genetic Markers of Susceptibility (CGEMS) breast cancer data [67], we found additional support implicating *SLCO1B1*. In CGEMS, five genotyped SNPs in *SLCO1B1 *(rs704166, rs852550, rs852549, rs7489119, and rs2306283) were associated with breast cancer risk with a *P *value of less than 0.05. These 5 SNPs, as imputed genotypes, were null in our dataset (data not shown), but this could be due to misclassification from imputation or false-positive associations across both CGEMS and our data. However, our findings and those of CGEMS, combined with the previous literature on the role of this gene in affecting estrone absorption, suggest that further investigation of the role of *SLCO1B1 *genetic variation and its interaction with EPT on breast cancer risk is warranted.

The strengths of this study include the systematic investigation of a large number of hormone metabolism genes and the detailed information on HT use collected at baseline. A limitation of our study was the inability to genotype all tagging SNPs for several of the genes of interest, including *AKR1C4, ARSC*, and *CYP19A1*. Thus, we cannot exclude the possibility that the lack of associations for these loci was due to incomplete tagging. Overall, we had 80% statistical power to detect ORs ranging from 1.17 to 1.40 for SNPs with an MAF of 0.05 to 0.49 by using log-additive models and an alpha of 0.05. For the subset analyses among postmenopausal women, the minimum detectable OR ranged from 1.20 to 1.46 for SNPs with an MAF of at least 0.05. The statistical power to detect associations in premenopausal women or to detect interactions with menopausal status or HT use was limited. Another limitation of this study is that HT use status assessed at baseline may have changed during follow-up. The participation rates (donating biological specimens for this nested case-control study) among the potentially eligible cohort members were moderate (74% for cases and 61% for controls). However, it is unlikely that the participation was differential according to genotype and case status, and thus selection bias is unlikely to have influenced our findings.

## Conclusions

Common genetic variations in *SLCO1B1 *may be associated with breast cancer risk in postmenopausal women, particularly in EPT users. The known effects of variants in *SLCO1B1 *on estrogen metabolism suggest that further study of the role of *SLCO1B1 *is warranted.

## Abbreviations

BMI: body mass index; BPC3: Breast and Prostate Cancer Cohort Consortium; CEPH: Utah residents with ancestry from northern and western Europe; CGEMS: Cancer Genetic Markers of Susceptibility; CI: confidence interval; CPIC: Cancer Prevention Institute of California; E1S: estrone sulfate; EPT: estrogen progestin combined therapy; ET: estrogen therapy; HT: hormone therapy; MAF: minor allele frequency; OR: odds ratio; P_ACT_: *P *value adjusted for multiple correlated tests; SNP: single-nucleotide polymorphism; UCI: University of California at Irvine; USC: University of Southern California; UTR: untranslated region.

## Competing interests

The authors declare that they have no competing interests.

## Authors' contributions

EL performed the statistical analysis and drafted the manuscript. FS, JPL, and SLN participated in the statistical analysis and drafted the manuscript. PLH-R, HAC, and LB participated in the design of the nested case-control study, were responsible for specimen collection, and obtained grant funding for the study. KDH prepared analytic variables, including menopausal status and hormone therapy use. AZ participated in coordinating the specimen process and SNP selection. DVDB coordinated tagging SNP selection and performed the genotyping. GU participated in the design of the study and in SNP selection and supervised the analysis and manuscript preparation. All authors edited the manuscript and provided comments on the intellectual content and read and approved the final manuscript.

## Supplementary Material

Additional file 1 **Supplementary Table S1**. A word document of Supplementary Table S1. Click here for file

Additional file 2 **Supplementary Figure S1**. A word document of Supplementary Figure S1. Click here for file

Additional file 3 **Supplementary Table S2**. An excel document of Supplementary Table S2. Click here for file

Additional file 4 **Supplementary Table S3**. A word document of Supplementary Table S3. Click here for file
