# Supplementary material for: The association of polymorphisms in hormone metabolism pathway genes, menopausal hormone therapy, and breast cancer risk: a nested case-control study in the California Teachers Study cohort
Source: Breast Cancer Res. 2011 Apr 1;13(2):R37. doi: 10.1186/bcr2859 (PMC3219200; doi:10.1186/bcr2859)
Supplement: Additional file 1 — Supplementary Table S1. A word document of Supplementary Table S1. [file bcr2859-S1.DOCX]

Supplementary Table 1. Proportion of SNPs in Hapmap dataset of European ancestry (release 24, Build 36) that are captured with pairwise r^2^≥0.80 by the tagging SNPs that were genotyped successfully (passing the quality control) in this study

| **Gene (chromosome)** | **Gene names** | **location** | **Tagging SNPs selected^‡^** | **Methods to select tagging SNPs^¶^** | **Total number of genotyped SNPs** | **Number of genotyped SNPs that are available in Hapmap r24** | **Hapmap SNPs in each gene region^*^ captured by the genotyped tagSNPs with minimum pairwise r^2^≥0.80** | **Number of genotyped SNPs that are not available in Hapmap r24** |
| --- | --- | --- | --- | --- | --- | --- | --- | --- |
| AKR1C4/HSD3A1 (10) | aldo-keto reductase family 1, member C4 / 3-alpha hydroxysteroid dehydrogenase, type I | 5,228,798 - 5,250,910 | No |  | 1 |  | - |  |
| ARSC/STS (X) | steroid sulfatase (microsomal), isozyme S / arylsulfatase C | 7,147,497 - 7,282,680 | No |  | 2 |  | - |  |
| COMT (22) | catechol-O-methyltransferase | 18,309,309 - 18,336,528 | Yes | Snagger | 20 | 19 | 88% (53 of 60) | 1 |
| CYP11A (15) | cytochrome P450, family 11, subfamily A, polypeptide 1 | 72,417,157 - 72,447,020 | Yes | Snagger | 12 | 12 | 95% (42 of 44) | 0 |
| CYP19A1 | cytochrome P450, family 19, subfamily A, polypeptide 1 |  | No |  | 2 |  | - |  |
| CYP1A1;CYP1A2 (15) | cytochrome P450, family 1, subfamily A, polypeptide 1; cytochrome P450, family 1, subfamily A, polypeptide 2 | [CYP1A1]  72,798,944 - 72,804,930  [CYP1A2]  72,828,237 - 72,835,994 | Yes | Snagger | 5 | 4 | 76% (10 of 13) | 1 |
| CYP1B1 (2) | cytochrome P450, family 1, subfamily B, polypeptide 1 | 38,148,250 - 38,156,796 | Yes | Snagger | 16 | 14 | 95% (43 of 45) | 2 |
| CYP21A2 (6) | cytochrome P450, family 21, subfamily A, polypeptide 2 | 32,114,061 - 32,117,398 | Yes | Snagger | 2 | 2 | 28% (2 of 7) | 0 |
| CYP2C9 (10) | cytochrome P450, family 2, subfamily C, polypeptide 9 | 96,688,430 - 96,739,137 | Yes | Snagger | 16 | 16 | 95% (65 of 68) | 0 |
| CYP3A4 (7) | cytochrome P450, family 3, subfamily A, polypeptide 4 | 99,192,540 - 99,219,744 | Yes | Snagger | 3 | 3 | 71% (5 of 7) | 0 |
| HSD17B1 (17) | hydroxysteroid (17-beta) dehydrogenase 1 | 37,957,510 - 37,960,757 | Yes | Snagger | 3 | 3 | 80% (8 of 10) | 0 |
| HSD17B2 (16) | hydroxysteroid (17-beta) dehydrogenase 2 | 80,626,364 - 80,689,638 | Yes | Snagger | 24 | 24 | 94% (67 of 71) | 0 |
| HSD17B4 (5) | hydroxysteroid (17-beta) dehydrogenase 4 | 118,816,124 - 118,905,923 | Yes | TagSNP (BPC3) | 24 | 24 | 97% (86 of 87) | 0 |
| HSD17B5/AKR1C3 (10) | hydroxysteroid (17-beta) dehydrogenase 5 / aldo-keto reductase family 1, member C3 | 5,126,568 - 5,139,876 | Yes | TagSNP (BPC3) | 38 | 36 | 96% (127 of 131) | 2 |
| HSD3B1 (1) | hydroxy-delta-5-steroid dehydrogenase, 3 beta- and steroid delta-isomerase 1 | 119,851,349 - 119,859,204 | Yes | TagSNP (BPC3) | 4 | 4 | 87% (14 of 16) | 0 |
| HSD3B2 (1) | hydroxy-delta-5-steroid dehydrogenase, 3 beta- and steroid delta-isomerase 2 | 119,759,296 - 119,767,172 | Yes | TagSNP (BPC3) | 10 | 10 | 100% (34 of 34) | 0 |
| SLCO1B1/SLC21A6 (12) | solute carrier organic anion transporter family, member 1B1 / solute carrier family 21 (organic anion transporter), member 6 | 21,175,403 - 21,283,995 | Yes | Snagger | 38 | 38 | 96% (154 of 160) | 0 |
| SRD5A1 (5) | steroid-5-alpha-reductase, alpha polypeptide 1 | 6,686,500 - 6,722,673 | Yes | TagSNP (BPC3) | 26 | 18 | 72% (62 of 86) | 8 |
| SULT1A1;SULT1A2 (16) | sulfotransferase family, cytosolic, 1A, phenol-preferring, member 1; sulfotransferase family, cytosolic, 1A, phenol-preferring, member 2 | [SULT1A1 v1]  28,524,419 - 28,528,858  [SULT1A2 v1]  28,510,767 - 28,515,892 | Yes | Snagger | 6 | 5 | 71% (10 of 14) | 1 |
| SULT1E1 (4) | sulfotransferase family 1E, estrogen-preferring, member 1 | 70,741,520 - 70,760,459 | Yes | Snagger | 18 | 18 | 93% (43 of 46) | 0 |
| UGT1A8 (2) | UDP glucuronosyltransferase 1 family, polypeptide A8 | 234,191,030 - 234,346,684 | Yes | Snagger | 42 | 42 | 93% (304 of 326) | 0 |
| UGT2B7 (4) | UDP glucuronosyltransferase 2 family, polypeptide B7 | 69,996,814 - 70,013,293 | Yes | Snagger | 5 | 5 | 96% (54 of 56) | 0 |

**^‡^** For *CYP19A*, we selected two SNPs shown to be associated with circulating estrogen levels through a systematic haplotype analyses (see reference [13]). For CYP21A2, the coverage is low because the tagging SNPs we selected were removed due to technical problems in genotyping. For AKR1C4, we selected a SNP that changes amino acid sequences (rs17134592; Leu311Val). For ARSC, we selected a SNP located in the 5’ UTR (rs5933863) and another SNP located in the 3’ UTR (rs1131289).

* From 20kb upstream of the start of each gene to 10kb downstream of the end of each gene, except for HSD17B4 and HSD17B5/AKR1C3 where the gene region covered upto 30kb upstream of the start of each gene.

^¶^ Tagging SNPs were selected using Snagger. Tagging SNPs selected by BPC3 were chosen using TagSNP program.
