# Supplementary material for: The association of polymorphisms in hormone metabolism pathway genes, menopausal hormone therapy, and breast cancer risk: a nested case-control study in the California Teachers Study cohort
Source: Breast Cancer Res. 2011 Apr 1;13(2):R37. doi: 10.1186/bcr2859 (PMC3219200; doi:10.1186/bcr2859)
Supplement: Additional file 2 — Supplementary Figure S1. A word document of Supplementary Figure S1. [file bcr2859-S2.DOCX]

Supplementary Figure 1. QQ plot for association of 317 SNPs in hormone metabolism pathway genes with breast cancer risk in the overall analyses
