# Supplementary material for: The association of polymorphisms in hormone metabolism pathway genes, menopausal hormone therapy, and breast cancer risk: a nested case-control study in the California Teachers Study cohort
Source: Breast Cancer Res. 2011 Apr 1;13(2):R37. doi: 10.1186/bcr2859 (PMC3219200; doi:10.1186/bcr2859)
Supplement: Additional file 4 — Supplementary Table S3. A word document of Supplementary Table S3. [file bcr2859-S4.DOC]

Supplementary Table 3. Odds ratios (OR) and 95% confidence intervals (CI) based on dominant genetic model for SLCO1B1 SNPs that were associated with breast cancer risk in subgroup analyses of postmenopausal women who were using EPT at baseline^*^

|  |  |  | Never used HT |  |  |  | Using ET |  |  |  |  | Using EPT |  |  |
| --- | --- | --- | --- | --- | --- | --- | --- | --- | --- | --- | --- | --- | --- | --- |
| Gene | SNP | N^**^  (ww/wv+vv) | OR (95% CI) | P^‡^ |  | N^**^ (ww/wv+vv) | OR (95% CI) | P^‡^ | P_int_^†^ |  | N^**^ (ww/wv+vv) | OR (95% CI) | P^‡^ | P_int_^†^ |
| SLCO1B1 | rs4149013 | 114/12+1 148/20+1 | 0.71 (0.33-1.52) | 0.37 |  | 199/31+0  245/31+0 | 1.19 (0.69-2.03) | 0.54 | 0.51 |  | 393/71+1  373/27+1 | 2.43 (1.53-3.85) | 0.0002 | 0.018 |
| SLCO1B1 | rs976754 | 110/16+1  143/25+1 | 0.84 (0.43-1.64) | 0.60 |  | 198/32+0  246/31+0 | 1.21 (0.71-2.05) | 0.48 | 0.51 |  | 375/91+2  351/49+1 | 1.74 (1.20-2.54) | 0.004 | 0.11 |
| SLCO1B1 | rs11045777 | 86/35+6  104/56+8 | 0.77 (0.46-1.28) | 0.31 |  | 155/66+9  180/82+14 | 0.88 (0.60-1.28) | 0.50 | 0.42 |  | 327/131+10  248/134+19 | 0.71 (0.54-0.95) | 0.020 | 0.95 |
| SLCO1B1 | rs11045773 | 86/35+6  103/56+9 | 0.75 (0.45-1.25) | 0.27 |  | 155/66+9  181/81+14 | 0.89 (0.61-1.29) | 0.54 | 0.36 |  | 326/131+10  248/134+19 | 0.71 (0.54-0.95) | 0.020 | 0.86 |

Abbreviations: EPT, combined estrogen-progestin therapy; HT, hormone therapy; ET, estrogen-only therapy; SNP, single nucleotide polymorphism; ww, homozygous major allele carriers of each SNP; wv, heterozygous minor allele carriers of each SNP; vv, homozygous minor allele carriers of each SNP.

^*^ Based on conditional logistic regression models stratified by age group (within 5-yr age groups) and specimen collection centers (CPIC, USC, UCI), using dominant genetic models.

^**^ N of cases carrying 0, 1, and 2 copies of minor allele and N of controls carrying 0, 1, and 2 copies of minor allele, respectively.

^‡^ P values not corrected for multiple testing

^†^ P values for interaction with never HT users.
